# A fixed nitrous oxide/oxygen mixture as an analgesic for patients with postherpetic neuralgia: study protocol for a randomized controlled trial

**DOI:** 10.1186/s13063-020-04960-5

**Published:** 2021-01-06

**Authors:** Hai-Xiang Gao, Jun-Jun Zhang, Ning Liu, Yi Wang, Chun-Xiang Ma, Lu-Lu Gao, Qiang Liu, Ting-Ting Zhang, Yi-Ling Wang, Wen-Qiang Bao, Yu-Xiang Li

**Affiliations:** 1 School of Nursing, Ningxia Medical University, 1160 Sheng Li Street, Yinchuan, 750004 China; 2 Intensive Care Unit, The Second People’s Hospital of Yinchuan, 684 Bei Jing Street, Yinchuan, 750011 China; 3 Department of Pharmacology, Ningxia Medical University, 1160 Sheng Li Street, Yinchuan, 750004 China; 4 Pain Department, General Hospital of Ningxia Medical University, 804 Sheng Li Street, Yinchuan, 750004 China; 5 School of Public Health and Management, Ningxia Medical University, 1160 Sheng Li Street, Yinchuan, 750004 China; 6 School of Preclinical Medical Sciences, Ningxia Medical University, 1160 Sheng Li Street, Yinchuan, 750004 China; 7 Nursing Department, The First People’s Hospital of Yinchuan, 2 Li Qun Street, Yinchuan, 750004 China

**Keywords:** Postherpetic neuralgia, Severe acute pain, Analgesia, Nitrous oxide, Randomized control trial

## Abstract

**Background:**

The pain management of postherpetic neuralgia (PHN) remains a major challenge, with no immediate relief. Nitrous oxide/oxygen mixture has the advantages of quick analgesic effect and well-tolerated. The purpose of this study is to investigate the analgesic effect and safety of nitrous oxide/oxygen mixture in patients with PHN.

**Methods/design:**

This study is a single-center, two-group (1:1), randomized, placebo-controlled, double-blind clinical trial. A total of 42 patients with postherpetic neuralgia will be recruited and randomly divided into the intervention group and the control group. The control group will receive routine treatment plus oxygen, and the intervention group will receive routine treatment plus nitrous oxide/oxygen mixture. Data collectors, patients, and clinicians are all blind to the therapy. The outcomes of each group will be monitored at baseline (T0), 5 min (T1), and 15 min (T2) after the start of the therapy and at 5 min after the end of the therapy (T3). The primary outcome measure will be the pain intensity. Secondary outcomes included physiological parameters, adverse effects, patients’ acceptance of analgesia, and satisfaction from patients.

**Discussion:**

Previous studies have shown that nitrous oxide/oxygen mixture can effectively relieve cancer patients with breakthrough pain. This study will explore the analgesic effect of oxide/oxygen mixture on PHN. If beneficial to patients with PHN, it will contribute to the pain management of PHN.

**Trial registration:**

Chinese Clinical Trial Register ChiCTR1900023730. Registered on 9 June 2019

**Supplementary Information:**

The online version contains supplementary material available at 10.1186/s13063-020-04960-5.

## Background

Postherpetic neuralgia (PHN) is a major complication of herpes zoster (HZ), and the definition of PHN is arbitrary but involves pain that lasts for 90 days or more from the onset of the HZ [1]. PHN is more common; one study found that the overall incidence of PHN was 3.9–42.0/100,000 person-years [2]. Age is a major risk factor for PHN, with a 4% risk of developing PHN under age 50 and a 34% risk over age 80 [3]. The proportion of the world’s population over 60 is expected to double in the next 50 years [4]. With the rapid increase in life expectancy, the incidence of PHN may rise sharply [5].

Spontaneous pain is common in PHN and can be intermittent or persistent. Pain has a variety of qualities, such as burning, throbbing, aching, stabbing pain, or electric-shock-like pain. Besides, allodynia and hyperalgesia have also been reported [6, 7]. PHN seriously harms the physical, psychological, and daily activities of patients. Patients lack appetite, lose weight, have trouble sleeping, and are reluctant to communicate with others. Some even suffer from depression and anxiety [8]. PHN also has a serious negative impact on an individual’s work; one study found that 64% of 88 participants missed work and 76% were less productive on account of HZ and PHN [9].

As mentioned above, PHN not only is physical pain but also causes irreparable detriment to all aspects of the patients. Effective treatment for PHN is urgently needed. However, there is no cure for PHN, and the main goal of current treatment is pain relief [10, 11]. The Neuropathic Pain Special Interest Group of the International Association for the Study of Pain (NeuPSIG) proposed some suggestions for the treatment of PHN. They advised gabapentin, pregabalin, and tricyclic antidepressants (TCAs) as first-line therapy; 5% lidocaine as second-line treatment; and opioids as third-line treatment [12]. But even with the most effective drugs, less than 50% of patients experience significant reduction (> 50%) in pain [13]. Gabapentin and pregabalin analgesia work slowly and take weeks to titrate to the therapeutic dose [14, 15]. Patients receiving the lowest dose of gabapentin continued treatment for an average of 17 weeks. The average daily dose of pregabalin is 187 mg, the average maximum dose is 222 mg, and it takes an average of 30 days to reach the maximum dose [14]. TCAs show a relatively slow onset of action with potentially troublesome adverse effects, such as cognitive impairment, excessive sedation, and cardiotoxicity (myocardial ischemia and arrhythmia) [16–18]. Five percent lidocaine takes hours (≤ 4 h) to act as an analgesic, acting faster than gabapentin or pregabalin [19]. These drugs are the most commonly used drugs to relieve PHN pain in clinical practice, which require a relatively long treatment period and cannot quickly relieve patients’ severe acute pain. Strong opioids such as oxycodone and morphine are comparable to TCAs in reducing pain in PHN patients and can quickly relieve severe acute pain [20]. Presumably for these reasons, one study found that 21.6% of PHN patients frequently used opioids as first-line therapy [21]. But the current guidelines for treating PHN with opioids are controversial [16]. This is not only related to the side effects of opioids such as constipation, nausea, and sedation, but PHN patients treated with opioids also have a higher medical burden [21, 22]. Besides, any physician who prescribes opioids should assess each patient’s personal and family history of substance abuse. And prescribing people must commit to regular monitoring of patients’ urine drug tests and stop taking opioids if there are signs of abuse [22]. That is, not all patients with PHN can use opioids for pain relief. Therefore, there is an urgent need to find a treatment that can relieve severe acute pain in PHN patients instead of opioids.

Nitrous oxide/oxygen mixture has been shown to have a fast and safe analgesic effect. In 1799, Humphrey discovered that nitrous oxide had anesthetic and analgesic effects [23]. The nitrous oxide does not irritate the respiratory tract, has very low solubility in the blood, and does not bind to the protein [24]. Nitrous oxide plays an analgesic role by stimulating the beta endorphin system and antagonizing the *N*-methyl-d-aspartic acid receptor [25]. Studies have shown that 30% of nitrous oxide produces an analgesic effect equivalent to 10–15 mg of morphine [26, 27]. In clinical practice, nitrous oxide and oxygen are mixed in different proportions for anesthesia or analgesia [28]. The nitrous oxide/oxygen mixture is stored in a pre-mixed cylinder and can be inhaled through a facemask or nasal catheter for self-administered analgesic. When inhaling nitrous oxide/oxygen mixture for analgesia, the patient is conscious, and adverse reactions are uncommon [29]. Now, it has been used in many countries around the world to relieve a wide variety of pain, such as childbirth [30], dental procedures [31], burn dressing [32], cancer patients with breakthrough pain [33], lumbar punctures [34], and other painful conditions. Another advantage of nitrous oxide/oxygen mixture is that it works quickly and recovers quickly [35]. Also, nitrous oxide/oxygen mixture is easily controlled and does not require a professional anesthesiologist to operate [36]. Side effects were mild and quickly resolved after cessation of inhalation [29]. Taking these advantages into account, this study will be conducted to explore the analgesic efficacy and safety of a fixed nitrous oxide/oxygen mixture (Patent no. ZL 2013 1 0053336.X) on PHN through a randomized controlled trial.

This study hypothesizes that the inhalation of nitrous oxide/oxygen mixture will be able to relieve pain in patients with PHN. It will be possible to replace opioids if nitrous oxide/oxygen mixture can effectively and rapidly relieve pain in patients with PHN, thereby reducing the risk of opioid abuse and lightening the financial burden on patients.

## Methods/design

### Aim

The main aim of the study is to evaluate the analgesic effect of the fixed nitrous oxide/oxygen mixture on patients with PHN. There are three secondary aims of the study:

1. To evaluate the safety of the fixed nitrous oxide/oxygen mixture by monitoring patients’ physiological parameters and adverse effects
2. To determine the satisfaction of patients with the analgesic effect of the fixed nitrous oxide/oxygen mixture
3. To investigate the acceptance of patients to the fixed nitrous oxide/oxygen mixture analgesia

### Study design

**Fig. 1:**
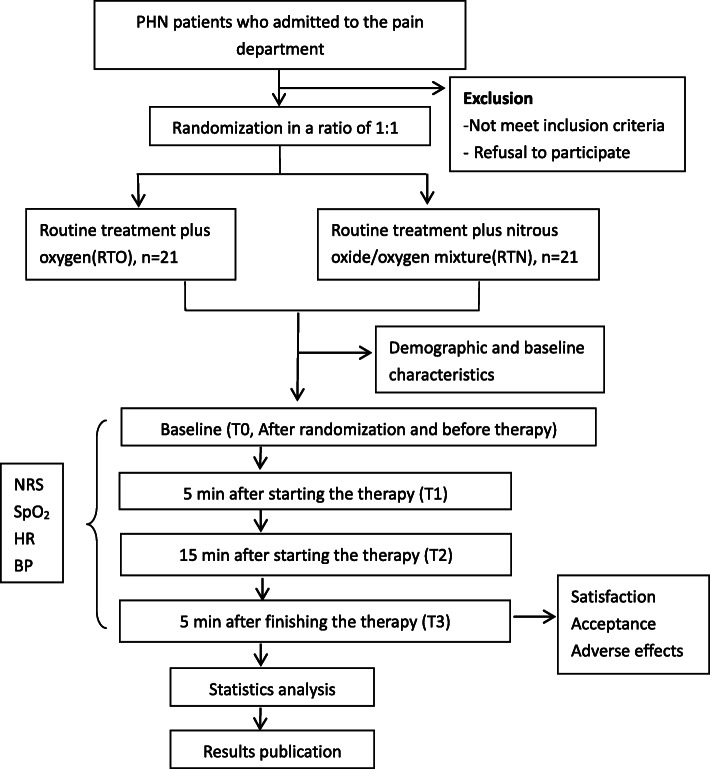
Study design framework. PHN postherpetic neuralgia, NRS numerical rating scale, SpO2 percutaneous oxygen saturation, HR heart rate, BP blood pressure, RTO routine treatment plus oxygen, RTN routine treatment plus nitrous oxide/oxygen mixture

This study is a single-center, two-group (1:1), randomized, placebo-controlled, double-blind clinical efficacy trial. We intend to investigate the analgesic effect and safety of nitrous oxide/oxygen mixture for PHN patients. Eligible participants will be randomly assigned to either the intervention group (nitrous oxide/oxygen mixture) or the control group (oxygen) based on computer-generated random numbers. The whole study design is shown in Fig. 1.

The protocol was approved by the Ethics Committee of the General Hospital of Ningxia Medical University (Reference No: 2018-373). The trial was registered with the Chinese Clinical Trial Registry (ChiCTR1900023730) on 9 June 2019. We adhered to Standard Protocol Items Recommendations for Interventional Trials (SPIRIT) for the design of the test (see Additional file 1).

### Study setting

This study will be conducted in the pain department of the General Hospital of Ningxia Medical University. This hospital is a tertiary hospital with functions of medical treatment, teaching, and scientific research. Approximately 100 PHN inpatients are admitted to the pain unit each year. This is sufficient to meet the sample size requirement.

### Study participants

All patients with postherpetic neuralgia admitted to the pain unit will be invited to participate in this study.

#### Inclusion criteria

Patients who meet the following inclusion criteria will be enrolled in the trial:

1. The patient met the Chinese PHN clinical diagnostic criteria (PHN is defined as pain persisting for ≥ 1 month after HZ rash healing [37]);
2. Over the age of 18;
3. Pain score ≥ 4 according to the Numerical Rating Scale (NRS); and
4. Volunteered to participate and signed the informed consent.

#### Exclusion criteria

Patients who have one of the following conditions are not allowed to participate in the trial:

1. Women who are pregnant;
2. The patient diagnosed with intestinal obstruction, air embolism, pneumothorax, and obstructive respiratory disease;
3. The patient’s clinical history includes epilepsy;
4. The patient suffers from otorhinolaryngologic diseases, such as sinusitis, middle ear disease, and eardrum transplantation;
5. Patient who is unable to report pain; and
6. Critically ill patients: (1) patients in intensive care, (2) patients after surgery, (3) patients with severe trauma or extensive burns, (4) patients with ventilator-assisted breathing, and (5) patients with life-threatening conditions requiring monitoring of vital signs.

### Recruitment

This study will be conducted in the pain department of the General Hospital of Ningxia Medical University. The number of PHN patients admitted to the pain department each year is enough to meet the sample size of this study. We will recruit participants through the pain department’s WeChat public platform, posters, and medical staff introductions. For patients who wish to participate in the study, researchers will elaborate on the purpose, methods, possible risks, and rights of participants. If the patient is fully aware of the study and agrees to participate and meets the inclusion criteria, the investigator will have the patient sign the informed consent.

### Randomization, allocation concealment, and blinding

Patients who meet the inclusion criteria will be randomly assigned to the intervention group and control group at a 1:1 allocation ratio after recruitment. The randomly assigned list will be computer-generated and performed by a statistician who is not involved in the trial and will be stored in a sealed, opaque envelope kept by the project manager. To ensure that the trial is double-blind, no investigator or patient will know about the randomization, only the project manager responsible for the gas distribution. The nitrous oxide/oxygen mixture and the oxygen will be supplied by the Ningfeng Oxygen Company, and the cylinder packaging of the two gases is identical. The letters A and B will be used to identify the two gases, and only the project manager will know what type of gas each letter represents. The project manager will distribute the gas according to the randomly assigned list.

### Interventions

A clinician is responsible for assessing the patient’s condition on the ward, determining whether the patient meets the inclusion criteria, and informing the patient of the purpose of the trial, methods, possible risks, and rights of participants. Patients who meet the inclusion criteria will sign an informed consent form after agreeing to participate in the trial. The project manager will group patients who agree to participate in the trial based on the randomly assigned list and escort the patient to the treatment room, where the trial will be conducted. All patients in the trial will receive routine treatment (oral pregabalin 75 mg twice daily). Patients in the intervention group will inhale nitrous oxide/oxygen mixture with a facemask, which is a visible intervention measure. To eliminate the influence of psychological factors on the experimental results of patients in the control group, patients in the control group will inhale oxygen by a facemask. Patients in the intervention group will receive routine treatment plus a fixed nitrous oxide/oxygen mixture (contains 65% nitrous oxide and 35% oxygen), while patients in the control group will receive routine treatment plus oxygen. Before inhaling the gas, the data collector will teach the patient to use the NRS to report pain intensity. Besides, the data collector will inform patients that they have the right to stop or quit the trial at any time if they experience discomfort during the study. Before the trial begins (T0), the data collector will record the patient’s demographic and baseline characteristics; assess the patient’s pain intensity; measure heart rate, blood pressure, and percutaneous oxygen saturation; and teach the patient to inhale gas using a facemask. Patients in both groups will inhale gas using an oral-nasal mask, which is disposable and has a one-way valve. According to our previous research, the gas intake will last for 15 min [33, 34]. When the data collector opens the valve of the cylinder and the patient puts on the mask to inhale the gas, the trial will start timing. The data collector will assess the patient’s pain intensity and measure blood pressure, heart rate, and percutaneous oxygen saturation at the beginning of the trial at 5 min (T1) and 15 min (T2). To assess the patient’s pain, the data collector will ask the patient: How bad is the pain now? Patients will point to the corresponding pain score with their fingers and without having to remove the facemask. Five minutes after the end of the trial (T3), in addition to collecting the data mentioned above, the data collector will ask patients about their satisfaction with pain relief and acceptance of analgesic methods. Throughout the trial, the data collector will closely observe and ask the patient whether there are adverse effects.

Nitrous oxide/oxygen mixture has been widely used for pain relief with mild adverse effects and quick recovery [29]. Adverse effects of nitrous oxide/oxygen mixture have been reported in the literature, including nausea, vomiting, dizziness, drowsiness, headache, hypotension, and oxygen desaturation [29]. These adverse reactions usually do not require medication and recover quickly [29]. If any adverse effects occur or the patient requests that the trial be discontinued, the data collector can determine whether the trial should continue. The reason for trial stopped will be recorded by the data collector. The project manager will accompany the patient to participate in the trial but does not involve in the collection of trial data. Only the project manager knows what kind of gas the patient is breathing. During the trial, the project manager will take treatment measures if the patient has adverse effects.

### Participant retention and withdrawal

To improve patients’ ability to adhere to the intervention, researchers will do the following strategies. First, the investigator will explain to the patient in detail the purpose of the study, the methodology, the possible risks, and the rights of the participants. Second, the researchers will closely monitor patients’ physiological parameters during the trial to ensure the safety of their lives. If patients have any discomfort during the trial, they will be available for medical treatment at any time. Third, patients who complete the trial will receive a free visit from a pain specialist. The investigator will create a chart to record the patient’s adherence to the trial. Patients can ask to withdraw from the trial at any time without affecting subsequent treatment. The data collector will record and analyze the reasons for the withdrawal.

### Data collection, management, and confidentiality

Members of the research team will be trained before the study. The training includes protocols for trial design (e.g., how to achieve double blindness), use of gas devices (e.g., use of facemask), data collection (e.g., how to assess a patient’s pain intensity), and experimental contingency planning (e.g., management of adverse effects in patients). Besides, data collectors and investigators will be fixed during the trial, and data will be double-entered to ensure accuracy. The Data Monitoring Committee (DMC) members will periodically review the database to improve data quality (see the “Data monitoring” section).

Patients will participate anonymously and their personal information will not be disclosed. The patient’s anonymous code will be randomly generated by the computer and kept by the project manager. The trial data will be stored in a folder on the computer that can only be accessed through password authentication. Researchers can check this data only with the authorization of the project manager. When the experiment is complete, the researchers will have access to the data for statistical analysis.

### Measurement

We will use a form to collect the baseline characteristics of patients, including age, gender, weight, height, education level, employment status, medical burden (no burden at all, basically no burden, some burden, heavy burden), location of pain, and course of the disease. Besides, the patients’ pain intensity, physiological parameters (blood pressure, heart rate, percutaneous oxygen saturation), patients’ acceptance of the analgesia, patients’ satisfaction with pain relief, and adverse effects will be recorded.

NRS will be used to assess the intensity of pain, ranging from 0 to 10, where 0 means “painless” and 10 means “worst pain imaginable” [38]. In most cases, clinicians and patients tend to use NRS to assess pain levels. Patients choose a number to represent their pain degree according to their feelings, which is easy to be understood by patients, is easy to use, and even can obtain scores in oral use, with high reliability and validity [39]. The reliability of the NRS has been proven to be moderate to high, with a maximum of 0.96. Besides, the convergence validity of NRS is 0.79 to 0.95 [40]. The patients’ physiological parameters will be monitored with an electronic sphygmomanometer (OMRON, HEM-7120) and oximeter (PC-60B). Pain intensity and the physiological parameters will be monitored simultaneously at baseline (T0), 5 min (T1), and 15 min (T2) after the trial began and at 5 min (T3) after the trial ended. Patients’ satisfaction with pain relief will be measured by a 5-point Likert scale (5, very satisfied; 4, satisfied; 3, uncertain; 2, dissatisfied; 1, very dissatisfied) [41]. The 5-point Likert scale is one of the Likert scales. Likert scale plays an important role in psychological research, which is the main method to measure attitude and personality. Besides, the Likert scale is easy to implement [42]. Furthermore, the data collectors will inquire into the patient’s acceptance of analgesia by asking the patient whether they would accept the gas inhalation to ease the pain (yes/no). Satisfaction and acceptance will be investigated at T3.

Starting with gas inhalation, the data collectors will carefully observe and record any adverse effects on the patient. The expected adverse effects of nitrous oxide/oxygen mixture therapy include nausea, vomiting, dizziness, drowsiness, headache, hypotension, and oxygen desaturation [29]. If any adverse effects occur, the study will be terminated immediately, the patient will be given oxygen, and the adverse effects will be fully reversible in 5 min [43]. Any adverse effects concerning the intervention will be recorded throughout the trial period.

### Outcome measure

#### Primary outcome measure

The primary outcome measure is pain intensity, which will be measured by NRS. The data collector will assess pain intensity at baseline, T1, T2, and T3. Before trial, patients will be taught to use the NRS to assess the pain intensity by pointing out the appropriate number (range = 0 to 10), without having to take off the facemask, thus ensuring continued inhalation of the gas.

#### Secondary outcome measures

The following are the secondary outcomes:

1. Patients’ satisfaction with pain relief
2. Patients’ acceptance of analgesia
3. Adverse effects
4. Physiological parameters (blood pressure, heart rate, percutaneous oxygen saturation)

**Fig. 2:**
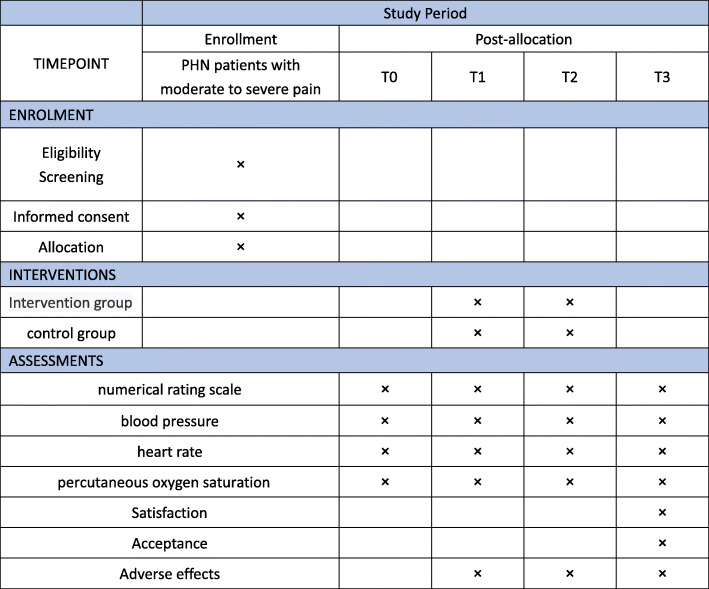
SPIRIT figure: schedule of enrolment, interventions, and assessments. PHN postherpetic neuralgia, T0 baseline, T1 5 min after starting the therapy, T2 15 min after starting the therapy, T3 5 min after finishing the therapy

Data collectors will collect data on patients’ satisfaction with pain relief, patients’ acceptance of analgesia, and adverse effects at T3. Patients’ physiological parameters will be collected at baseline, T1, T2, and T3 (Fig. 2).

### Data monitoring

The DMC will be set up at the same time the research project is identified. The DMC includes a pain management specialist, a chief pharmacist, a chief nurse, and a statistical specialist who chairs the DMC. During the implementation of the study, members of the DMC will review the original test data every month, check the standardization of the study, ensure the completeness and accuracy of the data, and guarantee the credibility and dependability of the study results. If the study is not performed as planned, the DMC will make corrective recommendations or stop the study. Any deviations from the trial will be recorded in the breach report form, and the study team will discuss and propose a modification plan. Update clinical trial registration information as necessary.

### Data storage

Paper material of the original test data will be stored in a locked filing cabinet in the pain department office of the General Hospital of Ningxia Medical University. All data will be input into the computer by two researchers, and access passwords will be set to keep the data secure. Personally identifiable subject information related to the data will be replaced with anonymous numbers. In order to ensure the quality of data, an independent clinical research assistant will review the original data every month to check whether the data is correct and complete. The authors will follow the guidelines recommended by the International Committee of Medical Journal Editors (ICMJE).

### Sample size calculation

We performed a pilot trial to calculate the sample size. According to our previous study on breakthrough pain [44], the sample size is calculated based on the pain intensity of patients, because the pain intensity is a primary outcome measure. Ducassé et al. [43] conducted a study on the analgesic effects of pre-mixed nitrous oxide and oxygen on patients with out-of-hospital trauma. The study confirmed that pain intensity in the intervention group (nitrous oxide/oxygen mixture) was significantly lower than that in the control group (medical air) at 5 min after administration (*p* < 0.001). Therefore, we used the pain intensity recorded at T1 as a reference in calculating the sample size. Our pilot trial involved 14 patients, with a 1:1 ratio between the intervention group and the control group. The sample size of this study was calculated by the following formula:

$$ {n}_1={n}_2=2{\left[\frac{\left({u}_{\alpha }+{u}_{\beta}\right)}{\raisebox{1ex}{$\delta $}\!\left/ \!\raisebox{-1ex}{$\sigma $}\right.}\right]}^2+\frac{1}{4}{u}_{\alpha}^2 $$

where *n*_1_ and *n*_2_ represent the sample sizes of each group. ***δ*** is the difference value between the two population means. ***σ*** represents the population standard deviation. According to the pilot trial, the pain severity during the test reported by all control group patients (*n* = 7) revealed a mean (SD) of 6.71 (1.28) out of 10. Patients (*n* = 7) in the intervention group reported a mean (SD) of 4.50 (1.80). So we got *δ* = |*μ*_1_ − *μ*_2_| =2.21. The standard deviation of the entire sample (*n* = 14) was 1.93, which was ***σ*** =1.93. ***α*** represents a significance level of 5% for a two-sided test, ***u***_***α***_ = 1.96. ***β*** was the type II error, which was set to 0.1; thus, ***u***_***β***_ = 1.282. We substituted these data into the formula and calculated that *n*_1_ and *n*_2_ were 16.9. Therefore, the sample size of each group was about 17. Taking into account the 20% drop-out rate before the end of the study, we decided to enroll a total of 42 participants (21 per group).

### Statistical analysis

The test data will be analyzed by a statistical expert using SPSS version 22.0 (Chicago, IL, USA). The statistical expert is not involved in the experiment and is only responsible for the analysis of the data. We will use descriptive statistical methods to measure demographics and baseline clinical features. The difference in the primary outcome between the two groups will be examined using the *t test.* A rank sum test will be used to compare the satisfaction with pain relief between the two groups. A chi-square test will be used to analyze the patients’ acceptance of analgesia. Adverse effects will be tested by the chi-square test. Physiological parameters will be compared using the repeated measures analysis of variance. Besides the complete case analyses, multiple computations are used to replace the missing values in the resulting parameters. Data will be performed with an intention-to-treat analysis. All results are statistically significant with *p* < 0.05.

## Discussion

PHN is a potentially debilitating neuropathic pain that is often undertreated. Viral damage to central and peripheral nerves may be the cause of PHN pain, which may be spontaneous, intermittent, or chronic, with no pattern [17]. PHN often affects the elderly, who have poor immunity and suffer from a variety of diseases. A survey of elderly PHN patients showed that pain severely interfered with their normal lives. Thirty-nine percent of patients with mild pain said they had at least moderate depression. Patients with moderate to severe pain were 49% and 60%, respectively. Only 14% of patients reported being “very satisfied” or “fairly satisfied” with the medication for pain relief [45]. At present, clinicians pay more attention to the long-term treatment effect of PHN, ignoring the rapid relief of pain. Patients must endure pain because analgesics work slowly. Therefore, rapid relief of pain in patients with PHN is particularly important.

In a previous research, Liu et al. [33] investigated the analgesic effect of nitrous oxide/oxygen mixture on cancer patients with breakthrough pain. The results showed that the pain intensity of patients who were treated with nitrous oxide/oxygen mixture decreased significantly at 5 min after the start of treatment (2.8 ± 1.3 versus 5.5 ± 1.2, *p* < 0.01). Furthermore, there were no serious adverse events associated with the nitrous oxide/oxygen mixture. The results of Liu et al.’s study indicated that the nitrous oxide/oxygen mixture can provide rapid and safe analgesia. Therefore, in this study, we will attempt to explore the analgesic efficacy and safety of a fixed nitrous oxide/oxygen mixture on PHN.

To date, this study is the first randomized controlled trial to investigate the effects of a nitrous oxide/oxygen mixture for pain relief in PHN patients. If this study proves that nitrous oxide/oxygen mixture is beneficial for PHN patients, it could be used as an emergency therapy for rapid relief of severe acute pain. PHN can be treated with nitrous oxide/oxygen mixture to compensate for the slow onset of systemic treatment and to reduce opioid use.

### Limitations

There are some limitations in this study. First, the definition of PHN used in this study is different from other studies because this study is conducted in a hospital in China, so the diagnostic criteria of Chinese postherpetic neuralgia are adopted (pain persisting for ≥ 1 month after HZ rash healing). The results of this study should be analyzed and discussed with the related research on postherpetic neuralgia. Second, this study is a single-center study with relatively limited research objects, which cannot fully represent the overall level. In the future, we will conduct multi-center research.

### Trial status

Recruitment of patients began on 8 October 2019. At the time of manuscript submission, the recruitment for this study is ongoing. Due to the prevalence of COVID-19, completion of this trial has been delayed and is expected to be completed until April 2021, originally scheduled for August 2020. At present, the trial has already recruited 20 participants. This is protocol version 3.0, dated 22 July 2019.

## Supplementary Information

**Additional file 1.** SPIRIT 2013 Checklist: Recommended items to address in a clinical trial protocol and related documents.

## Data Availability

After this study is completed, the final dataset and statistical codes will be accessible from the corresponding authors on reasonable request, except for patients’ personal information. The results will be published in peer-reviewed journals. Findings will be shared with the academic community, policymakers, and the general public.

## Abbreviations

PHN: Postherpetic neuralgia
HZ: Herpes zoster
NeuPSIG: The Neuropathic Pain Special Interest Group of the International Association for the Study of Pain
TCAs: Tricyclic antidepressants
AAN: American Academy of Neurology
SPIRIT: Standard Protocol Items Recommendations for Interventional Trials
NRS: Numerical Rating Scale
DMC: Data Monitoring Committee
ICMJE: International Committee of Medical Journal Editors
